# A New Method of Analysing Sprint, Deceleration, and Change of Direction Abilities in Trained Athletes

**DOI:** 10.3390/sports14010036

**Published:** 2026-01-13

**Authors:** Gregory Gordon, Andrew Green

**Affiliations:** Department of Sport and Movement Studies, Faculty of Health Science, Doornfontein Campus, University of Johannesburg, Johannesburg 2028, South Africa; andrewg@uj.ac.za

**Keywords:** novel, multidirectional sprints, MDSA

## Abstract

In modern sports, straight-line sprinting alone is insufficient for assessing overall sprint performance, as athletes must also decelerate and change direction efficiently. Existing methods lack a single metric that integrates all abilities, enabling holistic assessment. This study aimed to develop a comprehensive and novel measurement of multidirectional sprinting ability. Fifty-four university athletes (21.0 ± 1.5 years; 69.6 ± 9.1 kg; 172.6 ± 7.8 cm) performed linear sprints, decelerations, and 45°, 90°, and 135° change of direction (COD) tests in both directions over 30 m. Sprint accelerations and decelerations were recorded using a Stalker ATS II radar gun, while COD times were measured with stationary time gates. Sprint velocities were used to generate a multidirectional sprint area (MDSA), which was divided into forward, backward, left, and right sections. The MDSA method is calculated as the area of the octagonal polygon created by plotting eight velocity vectors from different angles of sprints. Paired t-tests compared area differences across directions, and ANOVA tests were used to compare sporting codes and sex. The resulting model reported differences across sporting codes (*p* < 0.001), sex (*p* < 0.001), the total area value (*p* < 0.001), and total area percentage (*p* < 0.001). The results showed a significant difference between forward and backward accelerations (*p* < 0.001), but no significant difference between left and right movements (*p* = 0.244). The MDSA method offers a reliable, quantitative intra-session approach for assessing athletes’ multidirectional sprint abilities by calculating the octagonal area on the basis of velocity data. This holistic analysis identifies asymmetries and performance weaknesses, providing valuable insights for coaches.

## 1. Introduction

Sprint performance, particularly when the sport requires a frequent need to change velocity, is a crucial factor in both track and field events and team sports, making it a major focus in many training programmes [1,2,3]. Sprinting at high velocities generates large mechanical power outputs, which rely on the athlete’s neuromuscular and osteoarticular systems to produce significant force at high contraction velocities [3,4]. Maximal velocity and sprint performance are fundamental in team sports, where athletes must accelerate efficiently over various distances, making sprinting one of the most essential skills for optimal performance [1,2,3,5]. Sprinting performance constitutes a critical individual performance trait in competitive sports, underpinning an athlete’s capacity to meet the dynamic demands of match play [6]. Research consistently links this attribute to enhanced match performance and favourable competitive outcomes across various sporting contexts [7]. As such, the ability to execute high-frequency and maximal velocity sprints directly influences individual contributions to team success, making it a pivotal factor in athletic evaluation [8]. However, the specific demands of sprinting vary significantly across sporting codes and between athletes of differing abilities and sexes [9]. For instance, sports like Australian Football League (AFL) have emphasised linear sprint speed, with superior 20 m sprint times strongly predicting draft success [10], whereas football has prioritised explosive sprints (i.e., short bursts of high acceleration) preceding goals or assists during match play [11]. In contrast, rugby demands greater multidirectional sprint capacity due to its chaotic, contact-driven nature [7]. Additionally, sex-based differences further complicate sprint profiles, as male athletes typically exhibit greater sprint distances and intensities than female athletes, reflecting disparities in physiological capacities and match dynamics [12]. Thus, finding time-efficient and practical methods as well as quantifying and developing maximal sprint qualities to assist in improving sprint abilities are vital for improving performance [13].

Sprint performance is determined by various phases, each requiring different contributions to optimise overall performance [14]. Typically, in sprints longer than 30 m, the action involves three key phases, namely acceleration, maximal velocity, and deceleration, while shorter sprints may not reach maximal velocity and subsequently experience lower deceleration [15]. During the acceleration phase, greater net horizontal force and ground reaction forces (GRFs) are produced as acceleration increases [3]. Efficient deceleration is crucial in high-velocity movements to optimise match performance [16], and occurs after every sprint, regardless of speed [17]. In team sports, deceleration helps players avoid defenders or boundaries [17]. Interestingly, during match play in field team sports, events involving decelerations occur more frequently than accelerations [18]. Every sprint performed will begin with an acceleration and will be followed by a deceleration aimed at slowing the body down [17].

Team sports involve a variety of vital match-play-determining changes in velocity, both accelerating and decelerating, which may relate to the tactical and technical aspects of the match [19]. While acceleration training has been extensively researched [3,20,21], there is a lack of research on rapid deceleration at the end of sprints or prior to COD [19]. Due to this paucity of information, there is substantially less data on the most efficient and practical ways in developing deceleration ability for athletes [22]. Due to decelerations occurring more than accelerations in competition [23], there is a need for more understanding of the negative consequences of these actions as a result of the higher impact decelerations have on the athletes [18]. Thus, it is essential for sport practitioners to have an efficient method to be able to profile an athlete’s maximal horizontal linear acceleration and deceleration abilities to inform coaches on improving performance as well as injury prevention [19].

Deceleration and change of direction (COD) capabilities are critical match-determining factors and fundamental for success in team-based field and court sports, where athletes must frequently alter direction, velocity, and reaccelerate—unlike the linear sprinting required in track and field events—with effective COD involving deceleration, redirection, and reacceleration [17,24,25,26,27,28,29,30]. COD speed is influenced by multiple determinants, including linear speed, kinetic mechanics, deceleration strategy, and physical capacity, such as strength and neuromuscular control [28,31]. COD performance is vital to sports, enabling athletes to navigate dynamic situations effectively [32]. The demands of COD vary with turning angle, as sharper turns require greater deceleration and braking forces [33,34]. A 90° COD, for instance, demands higher peak forces but lower speed and acceleration than a 45° COD [33]. Energy expenditure also rises with sharper angles, likely due to increased effort to accelerate from a lower velocity post-deceleration [33]. Therefore, it has been suggested that a holistic analysis of COD ability should involve testing multiple angles [35].

The purpose of this study was to create a new holistic method to analyse a player’s full sprinting ability including maximal linear sprint, COD, and deceleration. This aims to fill a gap for a method in which a sports practitioner can compare and analyse an athlete’s full multidirectional sprint performance. Currently, there is no current method which can assess an athlete’s comprehensive multidirectional sprinting ability. Additionally, this research set out to assess the method for sensitivity across sporting codes and sexes. Furthermore, the objective of determining the ability to quantify directional differences such as forward vs. backward and right vs. left in multidirectional sprints. It is hypothesised, in the novel multidirectional sprint area (MDSA) method, that left and right directional MDSA segments will be non-significant due to balanced bilateral demands in team sports.

## 2. Materials and Methods

### 2.1. Study Design and Participants

This study has a cross-sectional research design, collecting objective data from various tests in order to determine the relationship between variables. The testing was performed during the in season of the athletes’ competition schedule.

A sample of 54 first team university-level athletes (age: 20.54 ± 1.49 years; mass: 69.6 ± 9.05 kg; height: 172.6 ± 7.76 cm) was recruited from various sporting codes. The sample included 39 male participants (age: 20.64 ± 1.56 years; mass: 70.84 ± 9.1 kg; height: 174.92 ± 9.1 cm) and 15 female participants (age: 20.27 ± 1.28 years; mass: 66.33 ± 8.35 kg; height: 166.53 ± 5.46 cm). Informed consent was obtained prior to the testing of all participants. All participants were required to sign consent forms prior to commencing this research. Institutional ethical approval was granted and obtained for the research project (REC-1359-2022).

The participants were split into different groups according to their sport codes, namely football (*n* = 26), rugby (*n* = 17), and netball (*n* = 11) as well as their sex. Power analysis using the current sporting code distributions and a moderate effect (partial eta = 0.25) yielded a power of 0.966. The analysed data compared each of these and used an overall median to compare with each group. These sporting codes were selected due to their high demands for multidirectional movement and the inherent variability in sprinting requirements across playing positions, making them ideal for evaluating the holistic MDSA metric.

### 2.2. Data Collection

Data collection for the sprint and COD tests was conducted over two consecutive days on a natural grass field to capture the athletes’ multidirectional sprint performance while ensuring adequate recovery and minimising fatigue effects. The weather and environment conditions on both days were similar to ensure consistent conditions for testing. On the first day, linear sprint tests and deceleration assessments were performed, followed by COD tests on the second day. This two-day protocol was designed to allow a minimum of 24 h of rest between sessions, reducing the impact of cumulative fatigue on test reliability. Within each testing day, the sequence of tests was randomised for individual athletes to minimise order effects; however, each athlete adhered to their same personalised randomised order to ensure consistency and facilitate effective monitoring by the research team. Testing occurred under standardised environmental conditions, with athletes wearing their sport-specific footwear to replicate game-like scenarios and ensure consistent conditions for testing. Each participant completed a standardised warm-up prior to testing to optimise performance readiness which included a light jog, dynamic stretching, and short submaximal sprints.

#### 2.2.1. Linear Maximal Sprint

Sprint times were recorded over 15 m and 30 m distances using timing gates (Fusion Smartspeed, Fusion Sports, Coopers Plains, Queensland, Australia) set to a height of 0.8 m [36] as well as using a Stalker ATS II radar-gun (Stalker Pro, Radar Sales Inc., Richardson, TX, USA). Times were recorded to the nearest 0.01 s. Each sprint began from a stationary split stance position with the front foot positioned 30 cm behind the timing gate to prevent a false trigger. Participants were instructed to initiate their own start with no backwards step or “rocking motion” and to sprint as fast as possible. Each participant was allowed 3 trials interspersed by a passive recovery period of at least two minutes. The fastest 15 m split was used as a “criterion” time in the maximal horizontal deceleration test. Stalker ATS 5.0 software was used to record and analyse the data (Stalker Pro, Radar Sales Inc., Minneapolis, MN, USA).

#### 2.2.2. Maximal Deceleration Test

The maximal running deceleration ability test was performed with a 15 m maximal sprint followed by a deceleration. The same protocol at the start of this test, as well as using the Stalker ATS II radar gun, was applied during the maximal acceleration test. The participants were instructed to sprint maximally for the length of 15 m followed by an immediate deceleration. This deceleration led to a complete stop and then the participant began to backpedal back to the 15 m mark to ensure there was a clear moment to examine the event at which the participant stopped, signifying the end of the deceleration phase. The deceleration distance was then manually calculated from the 15 m mark to where the athlete’s velocity reached 0 m/s. Additionally, if the 15 m sprint time achieved was 5% greater than any of the maximal linear sprint tests, the trial was deemed as an unsuccessful trial, as the maximum-effort sprint was required to ensure consistency between tests. There was rest periods of a minimum of 3 min between each trial for the participant [18].

#### 2.2.3. Change of Direction

A COD task involves changing direction to a predefined direction after initially running forward. Six COD tests with the angles of the turns being 45°, 90° and 135° towards both the left and right side, totalling a 30 m sprint split into two. A 30 m distance was selected to allow sufficient acceleration to maximal velocity, reflecting match demands in team sports while enabling comparison across angles [15]. This test used timing gates to record the times for each section of the COD test. The participant began the sprint from a stationary split start, just as the maximal linear sprint and deceleration test began. The participant sprinted linearly for 15 m, then once they reached this point, marked with cones and timing gates, they began to change direction towards the next mark 15 m away. Once instructed to start the sprint, the participant ran as fast as they could, attempting to reach maximal velocity. As the first COD approached during the sprint, the participant decelerated to turn as efficiently as they could. The participant then continued to sprint as fast as they could through the timing gates. Each participant had three attempts for both a left and a right turn with adequate rest periods of a minimum of 3 min. The fastest attempt from the 15 m mark until the final 30 m mark for each participant was used for statistical analysis.

#### 2.2.4. Multidirectional Sprint Analysis

This new method aims at providing a holistic and universal approach to analysing an athlete’s multidirectional sprint ability. Athletes performed linear sprints, decelerations using the acceleration–deceleration ability (ADA) test, and three COD tests (45°, 90°, 135°) in both directions, from which the times were measured and velocities calculated. This equated to a total of 8 tests in equidistant directions using straight-line acceleration and decelerations as well as the different angles for the COD. The times for each athlete were recorded at both the 15 m mark as well as the 30 m mark. For the deceleration test, the 15 m mark was recorded as well as the point at which the athlete came to a complete stop. The linear sprint and CODs both comprised a full 30 m sprint, where, during the CODs, the athletes had to pivot at the 15 m mark.

Using the time taken to reach both marks during the athletes’ sprints (15 m and 30 m), the velocity for each was calculated using the following formula:

Velocity=Distance / Time

Velocity was derived from this calculation for all tests (linear sprint, deceleration, and COD) to ensure methodological consistency, as the radar gun could not be reliably applied to angled COD runs due to trajectory limitations. The velocities for each athlete were then plotted onto a radar graph to see the analysis of their multidirectional sprint performance, while also being able to compare with another athlete.

**Figure 1:**
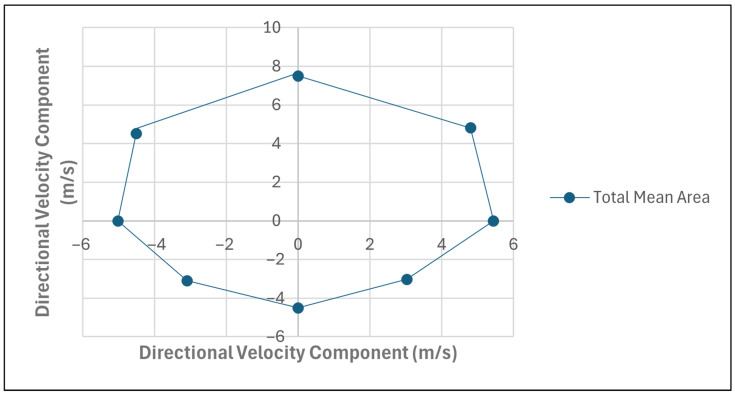
Scatter graph displaying the total mean area calculated for an athlete’s multidirectional sprint ability.

**Figure 2:**
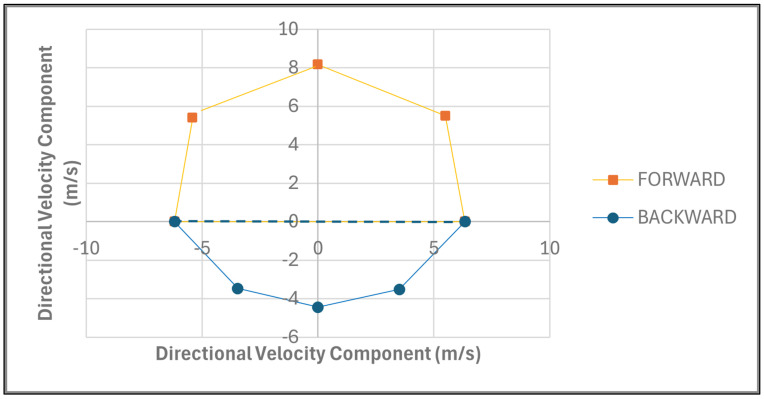
Scatter graph displaying the total mean area in the forward and backward directions calculated for an athlete’s multidirectional sprint ability.

**Figure 3:**
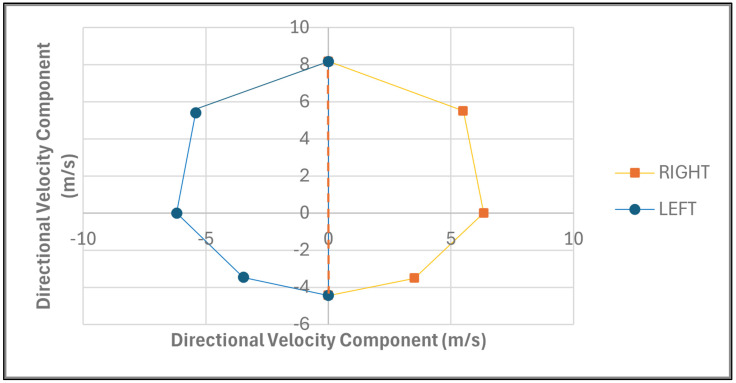
Scatter graph displaying the total mean area in the left and right directions calculated for an athlete’s multidirectional sprint ability.

The MDSA method was developed to quantify athletes’ multidirectional sprint ability across eight directional angles (0°, 45°, 90°, 135°, 180°, 225°, 270°, and 315°), corresponding to forward, backward, left, and right movements. The forward segment included the angles 0°, 45°, 90°, 270°, and 315°, while the backward segment included 90°, 135°, 180°, 225°, and 270°. Furthermore, the right segment included the angles of 0°, 45°, 90°, 135°, and 180° and the left segment included 0°, 180°, 225°, 270°, and 315°. Sprint velocities for each direction, derived from 15 m to 30 m tests, were used to calculate the x and y coordinates, based on their angular components. Velocity was selected to use as a metric due to it being a measurable value rather than one which is required to be calculated. These coordinates were plotted on a scatter graph (Figure 1), with the resulting polygon’s area computed using the shoelace formula (0.5|∑(x_i_ × y(i + 1) − x(i + 1) × y_i_)|) to represent the athlete’s multidirectional sprint capacity. The graph was segmented into four views—forward, backward, right, and left—enabling a comprehensive analysis of directional performance. The area for each segment was calculated and expressed as a percentage of the total area (Figure 1), with comparisons between forward and backward (Figure 2) and between left and right directions (Figure 3) used to assess symmetry. This approach provides a holistic and practical framework for evaluating an athlete’s MDSA.

### 2.3. Statistical Analysis

Shapiro–Wilk tests were used to assess data normality and confirmed a non-parametric distribution. Consequently, data were reported as medians and interquartile ranges (IQRs). Athletes were grouped by sporting code and sex. A Kruskal–Wallis test was applied to compare performance across sporting codes, while a Mann–Whitney U test evaluated differences between sexes. To compare forward versus backward and right versus left areas, the Wilcoxon signed-rank test, which was used for the sexes, and Friedman’s two-way analysis were employed for the sporting codes. The significance level was set at *p* < 0.05, with Kendall’s W calculated as an effect size metric for the Friedman’s test to quantify the magnitude of differences across directional areas. All analyses were conducted using IBM SPSS Version 29.

Intra-session reliability of the sprint tests was assessed using the intraclass correlation coefficient (ICC) calculated from the three trials per test by SPSS (version 29.0, IBM Corp., Armonk, NY, USA). The standard error of measurement (SEM) was derived as SEM = SD × √(1 − ICC), where SD is the standard deviation of the scores across all trials. The minimum detectable change at 95% confidence (MDC_95_) was computed as MDC_95_ = SEM × 1.96 × √2, with relative values expressed as percentages of the mean. Effect sizes for Kruskal–Wallis tests (comparing sporting codes) were calculated as eta-squared (η^2^) = H/(n − 1), where H is the test statistic and n is the total sample size; for Mann–Whitney U tests (sex differences), effect sizes were computed as r = Z/√n, where Z is the standardised test statistic and n is the total sample size. Effect sizes were interpreted following Cohen [37]: small (η^2^ = 0.01 to <0.06; r = 0.10 to <0.30), moderate (η^2^ = 0.06 to <0.14; r = 0.30 to <0.50), and large (η^2^ ≥ 0.14; r ≥ 0.50).

## 3. Results

When comparing the times of the athletes during each multidirectional sprint, we can see that the median and interquartile range values of the CODs with a greater angle of change have a longer time than those of the lesser angled CODs as well as the straight-line sprint (Table 1). However, the median and IQR of the athletes’ velocities from the 15 m mark to the 30 m mark demonstrates that when the angle of the COD is greater, then the velocity is less than that of sprints with lesser angled CODs (Table 1).

### 3.1. Sporting Code MDSA Comparison

Differences across sporting codes are noted in Table 2 for all directional sprint metrics. Furthermore, netball reported the smallest total area value compared with football (*p* ≤ 0.001), while rugby also reported a significantly smaller total area value compared with football (*p* ≤ 0.001). In addition, significant differences were found between all area values in each direction between football and netball (*p* ≤ 0.001), as well as between football and rugby (*p* ≤ 0.001). However, there was no significant difference between the area percentage in all directions among these sporting codes. In addition, there was a significant difference between rugby and netball for the area percentage of the forward (*p* = 0.022) and backward area (*p* = 0.030), but no significant difference was found for the total area value (*p* = 0.265), all directional area values, and the area percentage of the left (*p* = 1.00) and right (*p* = 1.00).

### 3.2. Sex MDSA Comparison

The differences between the sexes were noted in Table 3 for all directional sprint metrics. When comparing between sexes, there was significant differences found between the total area value (*p* ≤ 0.001), all directional area values, and the right area percentage (*p* = 0.049). Subsequently, there was no significant difference found between the area percentage for forward (*p* = 0.900), backward (*p* = 0.885), and left (*p* = 0.084) values between sexes.

### 3.3. Combined MDSA Comparison

**Figure 4:**
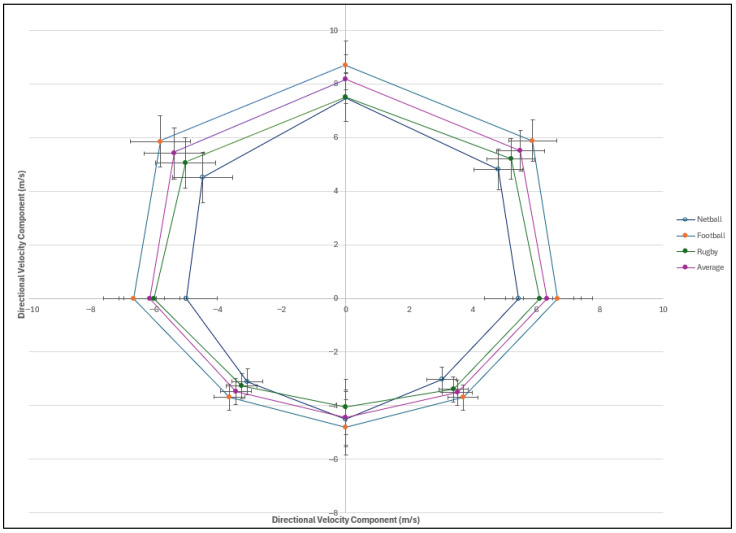
Scatter graph displaying the total mean areas and interquartile range for the sport codes calculated for an athlete’s multidirectional sprint ability.

The median values of each direction were also calculated due to the values being non-normally distributed and are displayed in Table 1. The median value of the total area is 124.59, while that for forward is 83.08 and that for backward is 37.55. Moreover, the median value for the area of the right direction is 61.84 and that for the left direction is 60.63. Thus, we can see greater area for the forward direction compared with the backward direction, as well as a slightly larger area for the left direction rather than the right direction for both the mean and median values (Figure 4).

Strong correlations were found between sprint velocity and total area, forward, and left and right areas (Table 4). Furthermore, moderate correlations were found between sprint velocity and the backward area (Table 4).

The median of the area percentages between forward (68.37%) and backward velocities (31.46%) showed a significant difference (*p* < 0.001). Conversely, there was no significant difference when comparing left and right velocities (*p* = 0.244). Sprints when running towards the left (49.61%) exhibited a smaller area of the velocity median percentage than that towards the right side (50.27%). The median of the velocity values of tests which moved in a forward direction is greater than that of the velocity of tests moving backwards (Table 1). The velocities of the CODs towards the right direction were remarkably consistent with that of the left direction (Table 1). Furthermore, the mean percentages of the areas were also greater for the forward direction (68.65%) compared with the backwards direction (30.65%). While not statistically significant, the right-side area (50.09%) was marginally larger than that of the left (49.20%).

This research found there are significant differences between sporting codes (Table 4) when considering both the median area values for total area (*p* < 0.001), forward (*p* < 0.001), backward (*p* < 0.001), left (*p* < 0.001), and right (*p* < 0.001). When considering the median area percentages, the forward percentage (*p* = 0.017), backward percentage (*p* = 0.033), and right percentage (*p* = 0.035) are found to be significant; however, the left percentage (*p* = 0.093) is found to have no significance. Additionally, when comparing the medians of the total area of the forward with the backward velocities, there is a significant difference (*p* < 0.001), while there is no significance when comparing the right- vs. left-sided areas of the sprint (*p* = 0.094).

The intra-session reliability of the MDSA and its component metrics was evaluated using data from the three trials per test. The ICCs were excellent, ranging from 0.85 to 0.92 for velocities across directions and 0.88 for the overall MDSA area, indicating high consistency in the measurements. The SEM for the total MDSA area was 1.10 units (relative SEM% = 0.88%), with values of 0.94 (1.13%) for forward, 0.22 (0.59%) for backward, 0.48 (0.78%) for left, and 0.65 (1.07%) for right areas, reflecting low absolute measurement error. For area percentages, SEM was 0.70 (1.03%) for forward and backward, and 0.60 (1.20%) for left and right. Among individual sprint velocities, SEM ranged from 0.03 m/s (0.40–0.70%) for the 45° and 90° COD tests to 0.05 m/s (0.92%) for the linear sprint, with the deceleration test at 0.03 m/s (0.60%). The MDC at 95% confidence for total MDSA was 4.32 units (MDC% = 3.47%), suggesting that changes exceeding this threshold represent true performance variations rather than measurement noise. The directional MDCs were 3.69 (4.44%) for forward, 0.87 (2.32%) for backward, 1.87 (3.02%) for left, and 2.57 (4.24%) for right areas, while the percentage MDCs were 2.76 (4.04%) for forward and backward and 2.36 (4.75%) for left and right. For velocities, MDCs ranged from 0.13 m/s (1.64–2.74%) to 0.20 m/s (3.68%) across tests, underscoring the method’s sensitivity to detect meaningful changes in multidirectional sprint performance.

Effect sizes for the Kruskal–Wallis comparisons across sporting codes were large for the total MDSA and its directional components (forward, backward, left, and right), indicating substantial practical significance in performance differences among football, rugby, and netball athletes. For the area percentages, effect sizes were moderate for the forward, backward, and left directions, but small for the right direction. Regarding sex differences, large effect sizes were observed for all area metrics, underscoring meaningful distinctions between males and females, whereas effect sizes for the percentage metrics were small across all directions.

## 4. Discussion

This study aimed to create a comprehensive evaluation of sprinting performance incorporating multiple directions of movement. To capture the data required for this novel method, an ordinary maximal linear sprint was used as well as COD tests and deceleration tests, which have been used previously and seen as reliable [22,38,39]. This initially gives us the relevant data to compare the medians of the athletes’ sprint abilities in multiple directions to see the relationships between the different types of sprints and the angles of each of the sprints. The MDSA method comparing sprints in multiple directions were plotted on a graph, with the area calculated to determine an athlete’s overall multidirectional sprinting ability. This approach, which is exclusively based on using previously reliable methods [4,18,33,35] to analyse different directions of sprints, approximately simulates real-life, multidirectional demands of field and court-based sports to provide insights into an athlete’s proficiency in multidirectional velocities, which may indirectly reflect transition efficiency.

The results indicate that as the COD angle increased, sprint times lengthened due to the greater deceleration and turning demands, which correspondingly reduced velocities—consistent with the interdependence of these metrics, as athletes initiated the second 15 m segment at progressively lower speeds. This is consistent with previous research which indicated that time of CODs increased as the angle of the turn became greater [29,40]. This increase in time and decrease in velocity when the angles are increased is likely due to the need to apply greater lateral forces in order to decelerate and reaccelerate at these larger turns [31]. Additionally, as the angle of the COD increased, there are changes in the vertical centre of mass displacement during the plant step, contact times, amount of deceleration steps, and the flexion and abduction of joints and the tibia angle during the plant step [29]. Consistent with the current study’s findings, Buchheit [40] observed that 45° COD tasks are most akin to straight-line sprinting, requiring less intense braking and propulsion forces compared with sharper angles. For different angles of COD, different strategies are needed to perform optimally; for example, more deceleration is required when pivoting around a larger angle [41].

To provide a full profile of athletes’ MDSA, each of the sprint tests were plotted onto a scatter graph to create a visual representation of their sprinting ability. This provides coaches with an easily accessible visual representation of their athletes’ multidirectional sprint ability, which can be compared with other athletes as well as with previous attempts at these tests. Subsequently, this method can also be used to compare an athlete’s MDSA with athletes in the same or different sport code, as well as athletes of the same or different sex. Moreover, the MDSA method can be used to report any asymmetrical differences in an athlete’s multidirectional sprint ability.

The area of the graph was calculated as a whole to provide an overall mathematical value to quantify their MDSA. This total area was then compared with the athletes’ area in all directions, namely forward, backward, right, and left. This study found the median value of the forward direction to be significantly larger than that of the backward direction. Logically, the forward direction will always have a greater area than that of the backward direction because the velocity running in a straight-line maximal sprint does not involve any COD and thus requires no need to decelerate and pivot to a new direction [31]. Likewise, running a COD at a 45° angle requires less deceleration, pivoting, and energy expenditure as compared with that of CODs with greater angles [33,34]. Additionally, the current study found that a sprint toward the right direction was found to be slightly larger than that toward the left direction. Previous studies found that athletes generally pivot quicker towards the side of their non-dominant leg [38,39]. However, those tests were only a 10 m COD, with the athletes changing direction after the first 5 m, whereas the current study used the velocity of the athlete at the point at which they reached the 15 m mark, where they were instructed to turn to a specific direction [38,39]. Although, in the current study, the right direction was greater than that of the left, this may not always be the case, as some athletes could be better at turning towards the left rather than to the right side, depending on their dominant leg [38,39], and thus this should also be viewed at an individual level to ensure that the athletes are being monitored for their own strengths. Thus, the MDSA method gives insight into discriminating any distinctions between forward and backward sprint performance, as well as any right and left variances during sprint performance. The MDSA could potentially identify directional asymmetries that reflect leg dominance preferences, rather than relying solely on left- or right-side designations, as in prior research, thereby facilitating more direct comparisons with those studies [38,39]. It should also be noted that in a training programme, coaches should not allow the athletes to favour one leg when pivoting at a COD to ensure that there is no significant asymmetry between the legs when turning [42].

A secondary objective of this study was to explore the differences in the multidirectional sprinting abilities of athletes between sporting codes and sexes. This study found that although there were significant differences for the different directions between sporting codes and sexes for all the median area values, there were no significant differences when comparing the percentages of the area between these groups. The observed differences in median area values across sporting codes and sexes likely stem from variations in absolute sprinting ability, physical conditioning, and sport-specific training adaptations. The stable percentage distributions across sporting codes and sexes, despite absolute differences in total MDSA values, may reflect inherent biomechanical constraints and fundamental movement patterns in multidirectional sprinting that exceed group-specific variations, as demonstrated by the consistent kinematic and mechanical profiles in the sprinting literature [12,31,43]. Different sports place varying demands on acceleration, deceleration, and COD abilities, leading to measurable disparities in sprint performance [26]. Similarly, differences in physical attributes such as muscle mass, strength, and power between the sexes could contribute to significant differences in absolute sprint performance, and these differences are seen to increase between sexes as the length of the sprints becomes larger [43]. However, the percentage of the area represents a normalised measure that accounts for the proportional distribution of performance across multiple directions rather than absolute values. Since percentage-based metrics emphasise the relative contribution of each direction to an athlete’s overall multidirectional sprint ability, they remain stable across different groups. This suggests that while athletes may differ in their total sprinting capacity, the way they distribute their sprint performance across directions remains consistent, potentially due to fundamental biomechanical constraints or training adaptations that apply universally across sports and sexes.

Several factors influence the area of the MDSA when considering the different velocities of the athletes. This research revealed that the areas for the different directions in the octagon were larger when higher velocities were achieved in the athletes’ performance. As such, higher velocities produced in the sprint tests will result in a larger MDSA area. This indicates that higher velocities produced in the sprint tests will result in a larger MDSA area. This is true for both the sprint and COD tests as well as the deceleration test. Furthermore, the velocity during the deceleration phase is approximately half the entry velocity at the 15 m mark; thus, athletes achieving higher entry speeds through superior initial acceleration will exhibit greater deceleration velocities, thereby decreasing the corresponding MDSA area segment. The observed pattern of large effect sizes for absolute MDSA measures across sporting codes and sexes, contrasted with small to moderate effects for relative percentages, underscores a key distinction in performance profiling. This disparity may be attributed to variations in muscular power, which contributes to larger absolute MDSA areas in groups exhibiting superior force production, such as males or football athletes in this study [4]. Furthermore, muscular power is dependent on body mass, as larger muscle volumes contribute to enhanced force output and sprint dynamics [44], thereby accounting for relative performance. Consequently, the normalised percentages reveal more consistent proportional distributions across codes and sexes by mitigating the influence of varying physical attributes and capacities.

This method offers the ability to compare and analyse an overall value of the area for the athletes’ MDSA, as well as breaking it down into percentages for each section of their multidirectional sprinting performance. By using velocity as the metric to calculate the area of their MDSA, it provides an independent measure without taking the athletes’ size and weight into account, which can be seen as making this a normalised measurement, thus providing the opportunity to compare athletes’ multidirectional sprint ability against both the same and different sporting codes and sex, as similarly emphasised in asymmetry analyses [45]. All athletes performed the same tests, showing that this task of creating an MDSA provides consistency in this method. By using area as the calculation, it removes any instances of subjective bias due to the objective data points that are used to make the calculation. Moreover, the consistent use of the area formula ensures statistical consistency, particularly if the data collection methods are standardised and thus repeatable, a principle echoed in multidirectional sprint protocols [46]. The area calculation within the MDSA holds potential for detecting performance changes, though this sensitivity requires empirical validation through dedicated analyses in future longitudinal studies. If the test is repeated and compared with previous attempts of an athlete, the velocity vector for a specific test may change, which will result in the total area as well as the area of a specific direction to change either positively or negatively. This can identify any incremental changes, which may assist coaches in identifying specific training regimen changes required for the athlete, aligning with findings on speed test interrelationships [47] and sprint training adaptations [48]. However, this method provides a metric that can be seen as a summary metric rather than providing specific details on each test. Therefore, this method should not replace other methods of analysis for sprint tests but should rather be used as an additional method to provide a summary of the athlete’s multidirectional sprint ability, complementing more detailed approaches like curvilinear or on-field assessments [46,49] and advanced biomechanical evaluations [50,51].

Nonetheless, this method can still be used to identify the strengths and weaknesses of an athlete’s multidirectional sprint ability. From a statistical perspective, the area serves as an aggregate measure of performance, as it uses multiple sprint tests and calculates it into a single value. Thus, the larger the total area, the better the overall sprinting ability of the athlete. Therefore, when comparing athletes’ MDSA values, a larger area may indicate greater overall multidirectional sprinting capacity, though this could be skewed by exceptional performance in specific directions (e.g., linear sprinting) rather than uniform proficiency across all tested components. Furthermore, the symmetry of the octagon can also represent the asymmetries and versatility of their multidirectional sprinting ability. A more symmetrical octagon for the athlete suggests that the athlete is similar in their ability to sprint in multiple directions. Conversely, a more irregular octagon suggests that there are clear weaknesses in their multidirectional sprinting ability. Although two athletes may have similar overall areas, it is important to consider the symmetry of the octagon because if the athlete’s octagon is irregular, then they may be very proficient in certain sprint tests but also may have weak points in certain other tests. Thus, it is important for a practitioner to always consider viewing an individual’s forward vs. backward and right vs. left areas to evaluate if there is an asymmetry in their performance and use this to improve their symmetry in multidirectional sprints. In addition, an athlete with a symmetrical octagon can be considered statistically more reliable in competitive settings, as their MDSA reveals that they have a wide range of movement types that are consistent throughout all directions. Furthermore, a process of using a dominant or non-dominant leg may give an alternative and more accurate method to provide a holistic MDSA rather than using right and left, which is a more general medium. This can provide coaches with the relevant information to change an athlete’s training programme in order to focus on the weaker areas of the athlete. Coaches can introduce appropriate multidirectional sprinting training programmes which can effectively improve their athletes’ multidirectional sprinting speed and power [52]. These training programmes do not have to directly focus on deceleration ability, even though this may also benefit the athlete, but complete multidirectional sprinting programmes can improve multidirectional speed, leg power, and dynamic stability [52].

### Limitations and Future Directions

A limitation could be found due to the relatively small sample that was used, and thus a larger sample of athletes should be used to find a norm of total area as well as the area of each direction for the multidirectional sprints. In comparison with males, there were fewer females who completed all tests, and this could be seen as a limitation, as a larger population of females may provide a better understanding of a female norm. With regards to the deceleration ADA test, this was the first time the athletes performed the test and with practice in this testing method, they may improve their ability to perform this test at their best. Thus, to ensure the athletes were comfortable with the testing methods, sufficient familiarisation and attempts were permitted. No indications were given to the athletes when turning during the COD tests as to whether to use a specific foot to turn to each side. Reliability was only assessed as intra-session reliability, and inter-session reliability could be used as well. The current MDSA method relies on velocity as a mass-independent metric, which overlooks the influence of body mass on momentum and change of direction performance; incorporating momentum calculations in future iterations could enhance the model’s applicability by accounting for these mass-related factors. This method has not been tested against match performance metrics, which would provide valuable insights into if the MDSA method relates to match performance. Due to large differences across the sporting codes, the sample average must be applied with caution. Additional studies within and across specific sporting codes are warranted to aid the development of normative values for the MDSA.

Testing additional angles to the COD tests could be used, which will provide more vectors for the polygon and provide an even more comprehensive view of an athlete’s MDSA. Introducing more sporting codes into the MDSA method may give a holistic view on multiple sporting codes and will give a more accurate norm for sport practitioners to work with. Comparing each athlete’s MDSA with regards to their positions within each sport to one another may create a holistic view for positions within sporting codes. Secondly, researchers could try to evaluate how this method’s outputs compare with other sprinting ability tests, such as strength and power tests, which have been shown to have direct comparisons with sprinting ability. Furthermore, by using MDSA to evaluate asymmetries of left vs. right as well as forward vs. backward directions and comparing these asymmetries with those of power tests such as a CMJ, drop jump, and Nordic curl. We could use the MDSA method to compare other sprinting metrics of the athletes apart from their velocity such as time, acceleration, and momentum of their sprints in multiple directions, as well as using the F-v profile of the athletes using this method. A longitudinal method could also be used to compare and evaluate how an athlete may improve or degrade their MDSA. Different sporting codes may require different ideal outcomes, and this could suggest using a sport-specific weighting. Due to the large quantity of decelerations that occur in a competition, an additional weight could be added to the aggregate measure. Additionally, future research and additional results will give a clearer view of whether a certain level of asymmetry will become problematic for an athlete’s multidirectional sprint ability. This method can be used for athletes coming back from injury to reach levels previously attained before the injury occurred.

## 5. Conclusions

The MDSA method to test an athlete’s multidirectional sprint performance can be seen as an efficient method due to the mathematical rigor behind the area calculations paired with an objective velocity measurement. Calculating the total area of the octagon may be seen as a powerful quantitative measure of an athlete’s objective overall sprinting ability and agility between both sporting codes and sexes. It can be used to provide a holistic view on one’s sprinting ability and give a mathematical and visual representation to provide the data to sports coaches and practitioners. This method assists with identifying asymmetries which expose weaknesses and irregularities in an athlete’s multidirectional sprint ability by analysing the visual representation of the octagon created, as well as comparing the area values and percentages. Although differences between the forward and backward directions were found, there were no significant differences between the right and left directions, which may differ when comparing individuals. However, the MDSA may not directly relate to other forms of other practices of testing multidirectional sprints. Thus, it is recommended to use this method in addition to other testing methods to have the full context of an athlete’s multidirectional sprint ability. Furthermore, this method can be seen as a summary metric as well as an additional method that can be used by practitioners to monitor the holistic multidirectional ability of an athlete. While the MDSA method should complement existing protocols, it is most valuable for broad athlete screening, long-term development monitoring, and group comparisons to highlight overall multidirectional sprint capacity and asymmetries, whereas individual component analysis is preferable for identifying technical deficits or guiding rehabilitation, ultimately enabling coaches to design targeted programmes for enhanced performance.

## Figures and Tables

**Table 1:** The median and the interquartile range (IQR) for each multidirectional sprint velocity and variables of the tests.

	Sprint (Forward)	Right 45	Right 90	Right 135	Deceleration	Left 135	Left 90	Left 45
0–15 Time (s)	2.77 (0.57)	2.41 (0.26)	2.49 (0.3)	2.56 (0.51)	3 (0.31)	2.55 (0.43)	2.5 (0.26)	2.41 (0.26)
15–30 Time (s)	1.83 (0.22)	1.9 (0.29)	2.31 (0.41)	2.93 (0.42)	1.48 (0.44)	2.99 (0.46)	2.37 (0.38)	1.9 (0.36)
0–15 Velocity (m/s)	5.42 (1.14)	6.24 (0.67)	6.02 (0.69)	5.86 (1.03)	5 (0.49)	5.88 (0.91)	6.01 (0.62)	6.22 (0.63)
15–30 Velocity (m/s)	8.22 (0.97)	7.92 (1.14)	6.51 (1.09)	5.12 (0.69)	4.24 (1.17)	5.03 (0.72)	6.34 (1)	7.92 (1.4)

**Table 2:** The median, interquartile range (IQR), *p*-values, and effect size for the median area values and percentages of all athletes compared by sporting code.

	Combined Sample (*n* = 54)	Football (*n* = 26)	Rugby (*n* = 17)	Netball (*n* = 11)	*p*-Value	Effect Size
Total area	124.59 (38.05)	132.47 (12.97)	108.39 (26.53)	87.56 (15.24)	<0.001	0.67
Forward	83.08 (25.49)	90.15 (6.98)	75.64 (14.5)	59.32 (4.63)	<0.001	0.66
Backward	37.55 (11.20)	40.35 (5.18)	32.75 (11.33)	28.24 (6.84)	<0.001	0.46
Right	61.84 (18.78)	64.49 (6.8)	55.21 (14.6)	45.32 (5.17)	<0.001	0.48
Left	60.63 (18.79)	66 (7.97)	53.18 (11.52)	42.24 (10.07)	<0.001	0.68
Forward %	68.37 (2.99)	68.03 (2.96)	70.11 (2.4)	67.84 (2.59)	0.017	0.12
Backward %	31.46 (3.57)	30.5 (3.98)	29.89 (2.4)	32.16 (2.59)	0.033	0.09
Right %	50.27 (2.19)	48.76 (2.14)	50.94 (2.69)	51.93 (4.04)	0.035	0.05
Left %	49.61 (2.05)	49.78 (2.03)	49.06 (2.69)	48.07 (4.04)	0.093	0.09

**Table 3:** The median, interquartile range (IQR), *p*-values, and effect size for the median area values and percentages of all athletes compared by sex.

	Combined Sample (*n* = 54)	Male (*n* = 39)	Female (*n* = 15)	*p*-Value	Effect Size
Total area	124.59 (38.05)	127.06 (15.19)	87.05 (17.76)	<0.001	0.59
Forward	83.08 (25.49)	87.86 (12.83)	59.9 (9.22)	<0.001	0.59
Backward	37.55 (11.20)	40.11 (7.03)	25.88 (9.63)	<0.001	0.44
Right	61.84 (18.78)	63.6 (8.4)	42.94 (6.05)	<0.001	0.50
Left	60.63 (18.79)	63.87 (10.13)	43.37 (10.02)	<0.001	0.58
Forward %	68.37 (2.99)	68.63 (2.45)	67.81 (8.75)	0.017	−0.04
Backward %	31.46 (3.57)	31.32 (2.74)	32.19 (8.75)	0.033	−0.04
Right %	50.27 (2.19)	50.08 (2.07)	51.38 (3.93)	0.035	0.04
Left %	49.61 (2.05)	49.77 (1.79)	48.62 (3.93)	0.093	0.02

**Table 4:** Correlation between sprint velocity and MDSA values.

	Total Area	Sprint Velocity
Sprint Velocity	0.829 *	- - -
Forward	0.969 *	0.827 *
Backward	0.802 *	0.581 *
Left	0.856 *	0.722 *
Right	0.984 *	0.786 *

* *p* < 0.001.

## Data Availability

The original contributions presented in this study are included in the article. Data are available upon reasonable request from the corresponding author.

## Abbreviations

The following abbreviations are used in this manuscript.

COD	Change of direction
MDSA	Multidirectional sprint area
ADA	Acceleration–deceleration ability
ICC	Intraclass correlation coefficient
SEM	Standard error of measurement
MDC	Minimum detectable change
IQR	Interquartile range
